# Metabolic flexibility of mitochondrial respiratory chain disorders predicted by computer modelling

**DOI:** 10.1016/j.mito.2016.09.003

**Published:** 2016-11

**Authors:** Łukasz P Zieliński, Anthony C Smith, Alexander G Smith, Alan J Robinson

**Affiliations:** a MRC Mitochondrial Biology Unit, Cambridge Biomedical Campus, Hills Road, Cambridge, CB2 0XY, UK; b University of Cambridge School of Clinical Medicine, Cambridge Biomedical Campus, Hills Road, Cambridge, CB2 0SP, UK

**Keywords:** Flux balance analysis, Electron transport chain, Mitochondrial disease, Metabolism

## Abstract

Mitochondrial respiratory chain dysfunction causes a variety of life-threatening diseases affecting about 1 in 4300 adults. These diseases are genetically heterogeneous, but have the same outcome; reduced activity of mitochondrial respiratory chain complexes causing decreased ATP production and potentially toxic accumulation of metabolites. Severity and tissue specificity of these effects varies between patients by unknown mechanisms and treatment options are limited. So far most research has focused on the complexes themselves, and the impact on overall cellular metabolism is largely unclear. To illustrate how computer modelling can be used to better understand the potential impact of these disorders and inspire new research directions and treatments, we simulated them using a computer model of human cardiomyocyte mitochondrial metabolism containing over 300 characterised reactions and transport steps with experimental parameters taken from the literature. Overall, simulations were consistent with patient symptoms, supporting their biological and medical significance. These simulations predicted: complex I deficiencies could be compensated using multiple pathways; complex II deficiencies had less metabolic flexibility due to impacting both the TCA cycle and the respiratory chain; and complex III and IV deficiencies caused greatest decreases in ATP production with metabolic consequences that parallel hypoxia. Our study demonstrates how results from computer models can be compared to a clinical phenotype and used as a tool for hypothesis generation for subsequent experimental testing. These simulations can enhance understanding of dysfunctional mitochondrial metabolism and suggest new avenues for research into treatment of mitochondrial disease and other areas of mitochondrial dysfunction.

## Highlights

• Mitochondrial diseases are life-threatening, but lack effective treatments.
• The impact of respiratory chain diseases on central metabolism is largely unclear.
• We built a computer model of mitochondrial and associated cytosolic metabolism.
• We simulated dysfunction of each complex of the mitochondrial respiratory chain.
• Simulations show different impacts to metabolism and suggest interventions.

## 1 Introduction

**Fig. 1:**
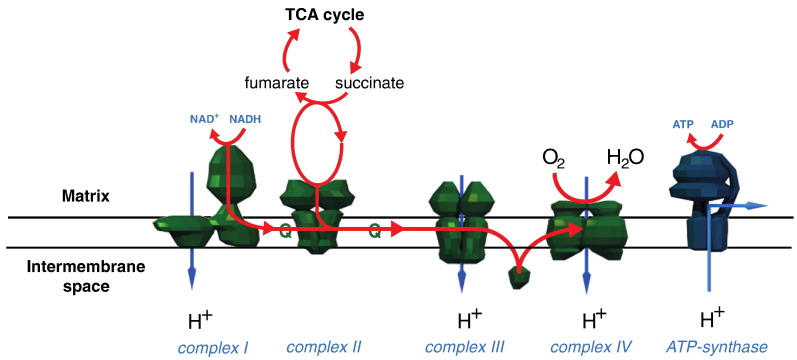
The complexes of the mitochondrial respiratory chain. The complexes of the respiratory chain are a major component of the mitochondrial inner membrane. Complexes I, III and IV pump protons across the mitochondrial inner membrane from the matrix into the intermembrane space. Proton pumping is achieved by coupling it to the energy released by the transfer of electrons down the chain from NADH and succinate to oxygen, to form water. The translocation of protons across the membrane creates a proton gradient that, in combination with the membrane potential, creates the proton motive force across the membrane. ATP synthase uses this proton motive force by coupling ATP generation to the transport of protons back across the membrane.

Mitochondrial respiratory chain disorders are the most common forms of mitochondrial disease and directly affect cellular ATP production (Fig. 1) (Smeitink et al., 2006). Isolated and combined respiratory chain disorders present in multiple ways (Pagniez-Mammeri et al., 2012a, Pagniez-Mammeri et al., 2012b, Mourmans et al., 1997, Bohm et al., 2006), with variable age of onset (Mourmans et al., 1997, Bohm et al., 2006, Taylor et al., 1996), and can be caused by mutations in structural or assembly genes of the constituent respiratory complexes. However, it is rarely clear how different mutations cause specific clinical phenotypes and affect enzyme activity (Pagniez-Mammeri et al., 2012a, Pagniez-Mammeri et al., 2012b, Ohlenbusch et al., 2012, Loeffen et al., 2000, Koene et al., 2012, Jain-Ghai et al., 2013, Sacconi et al., 2003).

Effective diagnosis of mitochondrial disorders is hampered by their wide genetic and metabolic heterogeneity, and most therapies focus on palliative care (Viscomi et al., 2015). These therapies include anti-epileptics, correction of metabolic acidosis, supplementation of respiratory chain cofactors or precursors (such as flavin nucleotides) and antioxidants. Ketogenic diets, used for seizure control in (mitochondrial) epilepsy, have also been suggested to promote energy production in failing mitochondria of patients with complex I deficiencies (Roestenberg et al., 2012, Kanabus et al., 2016, Laugel et al., 2007, Leung et al., 1998, Paoli et al., 2014). However the molecular rationale and effectiveness of these treatments are not established (Chinnery et al., 2006). Greater understanding of these disorders could improve patient diagnosis, survival rate, and the design of new targeted therapies, as well as anticipating potential side-effects of treatments.

A challenging aspect of mitochondrial disorders is studying their effect on overall cellular metabolism. Addressing this is non-trivial as metabolism is a complex network of interlinked reactions with interactions that can be unintuitive. Studies have identified perturbed metabolic pathways in dysfunctional mitochondrial by using transcriptomics and gene enrichment analyses (Falk et al., 2008, Zhang and Falk, 2014), but the application of large-scale metabolomics in mitochondrial diseases is uncommon (Shaham et al., 2010). However, new computational systems biology methods can aid analysis of experimental data and suggest testable hypotheses by incorporating this complexity, and we have previously modelled metabolic consequences on overall metabolism of ischemia (Chouchani et al., 2014) and enzyme deficiencies of the TCA cycle (Ashrafian et al., 2012, Smith and Robinson, 2011). Thus, we applied our computer model of human cardiomyocyte mitochondrial metabolism (Smith and Robinson, 2011) to predict changes occuring in respiratory chain disorders.

We simulated the metabolic networks using flux balance analysis (Orth et al., 2010) and demonstrate how simulations can generate hypotheses to identify new avenues for potential therapies and so improve understanding of respiratory chain disorders. The relevance of these simulations was demonstrated by the reproduction of patient metabolic phenotypes, including accumulation of organic acids and alanine. By using the simulations, we identified a range of metabolites and mechanisms predicted to lead to these phenotypes and to compensate for low respiratory chain activity. Our results suggest the ability to accommodate the metabolic impact of respiratory chain deficiencies varies with different respiratory complexes, and that the metabolic impact of complex III and IV deficiencies has parallels with hypoxia.

## 2 Materials and methods

### 2.1 Flux balance analysis of metabolic networks

We simulated metabolism of the metabolic network by using flux balance analysis (FBA), which calculates fluxes (flows) of metabolites through a network of biochemical reactions (Orth et al., 2010). FBA requires a number of assumptions and constraints to enable the calculation of fluxes. The major assumption of FBA is that fluxes are calculated at a pseudo steady state, and with conservation of mass requires the total rate of production and consumption of metabolites to be zero, although “sources” and “sinks” enable simulation of metabolite influx and efflux to the system. An impact of this assumption is that the NADH/NAD^+^ ratio was fixed in the simulations, i.e. the rate of NADH production was equal to its oxidation so that NADH and NAD^+^ are coupled. Thus in situations where the NADH/NAD^+^ ratio may be expected to change; the model will find pathways to circumvent this. For example, in scenarios where experimentally the NADH/NAD^+^ ratio may be expected to increase (e.g. complex I dysfunction), flux balance analysis will find alternative pathways that attempt to compensate for complex I dysfunction and reoxidise NADH. In FBA the fluxes around the network are constrained by reaction stoichiometry and directionality, as well as capacity constraints that specify upper and lower bounds on reaction fluxes and metabolite uptake. These constraints are defined from experimental information such as enzymatic activity, estimates of Gibbs free energies, or metabolite uptake rates such as glucose uptakes or oxygen consumption. Defining ranges allows the uncertainty of a flux value to be taken into account. But where the reaction flux is unknown, the default is unlimited flux in either direction. To find the flux distribution most likely to resemble that in vivo, an objective function is chosen that best matches the primary purpose of the system, and FBA is used to calculate the optimal fluxes that maximise this function. For the mitochondrial respiratory chain, we chose maximum ATP production to represent its primary purpose in energy generation. Unfortunately, the residual function of respiratory chain components is often reported only as enzyme activity. But, the relationship between enzyme activity and flux is usually complex and non-linear (Kacser and Burns, 1981). To account for this, we reduced the maximum flux through the respiratory chain complexes to different percentage values of the normal flux (0% (inhibited), 5%, 10%, 25%, 50% and 75%), and investigated how this affected maximum ATP production to gain a qualitative understanding of how different enzyme activities cause changes to metabolism.

FBA simulations were carried out by using MATLAB (Math Works, Inc., Natick, MA) with the COBRA Toolbox (Becker et al., 2007), and the linear programming solver GLPK (http://www.gnu.org/software/glpk). The simulations were visualized using Cytoscape (Lopes et al., 2010).

### 2.2 Model of human cardiomyocyte mitochondrial metabolism

Simulations were performed on an updated and expanded version of our earlier myocardial mitochondrial metabolic model, *i*AS253 (Smith and Robinson, 2011). The model includes the TCA cycle, glycolysis, pentose phosphate pathway, β-oxidation of fatty acids, and ketone body and amino acid degradation, and covers all parts of central metabolism (both cytosolic and mitochondrial) that contribute to the respiratory chain. As the availability of different metabolites varies considerably between the cytosol and mitochondrion, we manually evaluated the localisation evidence of enzymes involved in central metabolism to partition the reactions they catalyse between the cytosolic and mitochondrial compartments, by using MitoMiner, a mitochondrial proteomics database (Smith and Robinson, 2016). Localisation of enzymes to heart tissue was evaluated from mass spectrometry datasets of cardiac tissue stored in MitoMiner, as well as information from BRENDA (Chang et al., 2015), Human Protein Atlas (Uhlen et al., 2015) and the literature. The cytosolic and mitochondrial compartments were connected with 83 transport steps modelled on the known capabilities of mitochondrial transporters (such as proton coupling and substrate exchange). Constraints on reaction directionality were imposed based upon the literature, defined rules of reaction irreversibility (Ma and Zeng, 2003), estimates of thermodynamics using the group contribution method (Jankowski et al., 2008), and information from public metabolic databases (Chang et al., 2015, Romero et al., 2005, Kanehisa et al., 2014). Capacity constraints representing the import and export of metabolites (such as glucose, fatty acids, amino acids and oxygen) were taken from the *i*AS253 model (Smith and Robinson, 2011), which were originally derived from the literature. The model contained 277 metabolites, 227 mitochondrial matrix reactions, 76 cytosolic reactions, 83 transport steps between the mitochondrial and cytosolic compartments, and 84 boundary conditions representing inputs and outputs into the system. Thus in total there are over 300 reactions in the model. As with our previous model, no metabolite dead ends were present and all reactions were capable of having a flux. The model is available in SBML format (Supplementary File 1).

## 3 Results and discussion

To investigate possible metabolic consequences of respiratory chain disorders, we simulated their effect using our model of human cardiomyocyte mitochondrial metabolism. This is a useful cell type to simulate as it can use a large array of metabolites to generate ATP (as encapsulated in the model). This increases the likelihood of finding metabolic bypasses, and lacks specialised tissue specific reactions (such as found in kidney and liver) that may make any mechanisms found less generalizable. We applied Flux Balance Analysis (Orth et al., 2010) to simulate the flow of metabolites around the metabolic network, and to identify pathways that maximised ATP production, reflecting the primary purpose of the respiratory chain. ATP production also serves as a proxy for the proton motive force—a key indicator of mitochondrial health, but which cannot be modelled directly by the flux-based FBA method—as the pumping of protons is directly coupled to ATP synthesis in the model.

### 3.1 Simulation of basal metabolism in healthy cardiomyocytes

To establish baseline flux values as controls to compare against fluxes from simulations of respiratory chain disorders, we simulated a cardiomyocyte model under basal conditions. In this simulation (as reported previously (Smith and Robinson, 2011)) (Table S1): all respiratory chain complexes were active; metabolic fuels were in slight excess showing oxygen diffusion was the major limiting factor of their reaction fluxes; ATP production was 138.1 μmol/min/gDW; and acetyl-CoA was derived from: fatty acid β-oxidation (58%), glycolysis (36%), ketone body oxidation (5%) and amino acid oxidation (less than 2%). Amino acids—arginine, lysine, proline, aspartate, cysteine, isoleucine, valine, leucine and serine—were imported into the system, and ammonia exported.

### 3.2 Simulations of complex I deficiency

Complex I (NADH:ubiquinone reductase) deficiencies are the most common respiratory chain disorders, accounting for up to 40% of cases (Loeffen et al., 2000, Kirby et al., 1999). Mammalian complex I couples oxidation of NADH to reduction of ubiquinone; pumping protons across the inner mitochondrial membrane and contributing to the electrochemical gradient. Common presentations of complex I deficiency include cardiomyopathies, encephalopathies, leukodystrophies, liver disease and Leigh syndrome (Pagniez-Mammeri et al., 2012a, Pagniez-Mammeri et al., 2012b, Loeffen et al., 2000, von Kleist-Retzow et al., 1998), and complex I inhibition in glial cells is linked to Parkinson's disease (McNaught and Jenner, 2000). Complex I deficiency can cause accumulation in body tissues of metabolites such as lactate, alanine and various TCA cycle intermediates (Loeffen et al., 2000, Dionisi-Vici et al., 1997, Procaccio et al., 1999, Moreadith et al., 1984, Esteitie et al., 2005). Complex I deficiency hampers the most efficient mechanism of oxidising mitochondrial NADH and feeding electrons into the respiratory chain. Consequently in patients the NADH/NAD^+^ ratio increases, but for the TCA cycle and other enzymes to continue functioning, NADH must be recycled back to NAD^+^. Therefore for cells to compensate for complex I deficiency, they need to find alternative mechanisms to reoxidise the NADH, or reduce its production. In our model the NADH/NAD^+^ ratio is fixed, so in a similar manner either the simulations find NADH reoxidising pathways to compensate for the loss of complex I activity, or NADH production is reduced and consequently ATP synthesis is decreased.

To investigate how metabolism may compensate for complex I deficiency, we simulated inhibition of complex I to varying degrees and examined how ATP production was maintained. When a compensatory mechanism emerged, it was subsequently disabled to reveal further, albeit less efficient, mechanisms. In this way, we identified several pathways predicted by the model to maintain ATP production that share a common mechanism of transferring electrons from mitochondrial NADH to other redox couples—which then enter the respiratory chain via the quinone pool—thus regenerating NAD^+^ and bypassing dysfunctional complex I.

#### 3.2.1 Complex I deficiency: compensation by the glycerol phosphate shuttle and proline dehydrogenase

**Fig. 2:**
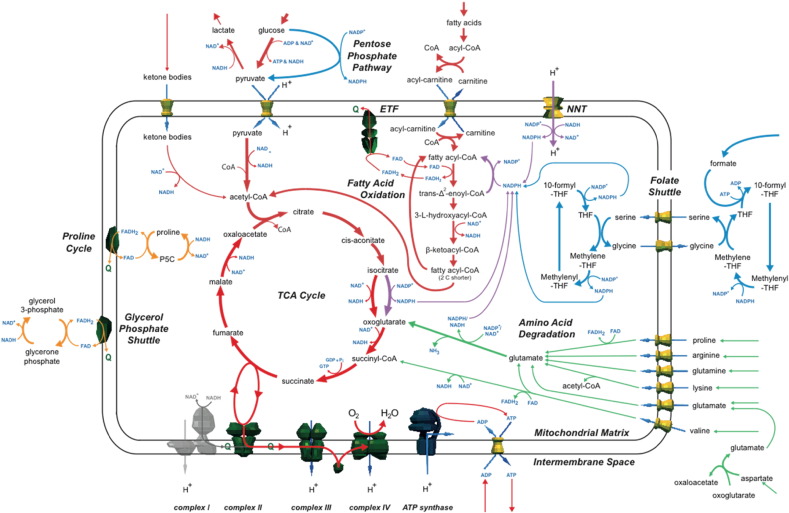
Summary of simulations on the effect of complex I deficiency on metabolism. As flux through complex I was inhibited in the simulations (grey), capacity to oxidise mitochondrial NADH was reduced, affecting the quinone pool, and decreasing ATP production. The model predicted several alternative pathways to compensate for the reduced capacity mitochondrial NADH oxidation and compensate for loss in ATP production. First, the activity of the malate aspartate shuttle was replaced by the glycerol phosphate shuttle, and mitochondrial NADH was oxidised by a proline cycle (orange). Second, glycolysis was reduced (although lactate efflux increased), whereas β-oxidation of fatty acids increased (red). Enzymes for the β-oxidation of fatty acids catalysed a cycle (red and purple) that oxidised mitochondrial NADPH and reduced the quinone pool via FADH_2_ and the ETF. Third, a hypothetical folate shuttle (blue) emerged that used THF metabolism and the pentose phosphate pathway to import NADPH to the mitochondrion.

Completely inhibiting reaction flux through complex I reduced ATP production from 138.1 to 95.6 μmol/min/gDW (Table S2); indicating substantial compensation for complex I inhibition, and corroborates accounts of short-term survival of patients with undetectable complex I activity (von Kleist-Retzow et al., 1998, Bugiani et al., 2004, Kirby et al., 2004). As flux through complex I was reduced, the first pathways to become inactive in the simulation were the metabolism of ketone bodies, serine and leucine. The metabolism of other metabolic fuels was uninterrupted, such as glucose, lactate, fatty acids and other branched chain amino acids. Two mechanisms in the simulation compensated for decreased mitochondrial NADH oxidation by dysfunctional complex I while still reducing the quinone pool (Fig. 2). First, the glycerol phosphate shuttle used cytosolic NADH (generated from glycolysis and the conversion of lactate to pyruvate). Second, mitochondrial NADH was oxidised by a cycle between proline dehydrogenase and pyrroline-5-carboxylate reductase. However, as neither of these mechanisms were proton-coupled, the efficiency of ATP production was reduced compared to the normal mechanisms. The significance of this predicted proline cycle in complex I deficiency is unknown and its activity is undocumented, but proline dehydrogenase contributes to the regulation of cellular redox state and is induced in a variety of cancers (Phang et al., 2010). Thus, the model generates the hypothesis that this mechanism may be active and compensate complex I deficiency when NADH:NAD^+^ ratio is high.

#### 3.2.2 Complex I deficiency: lactate acidosis and compensation by β-oxidation of fatty acids

Limiting the reaction flux through the glycerol phosphate shuttle had a negligible effect on maximum ATP production (Table S3), suggesting the model found further compensatory mechanisms. However, this inhibition disfavoured the production of cytosolic NADH from lactate and glucose metabolism, and resulted in the efflux of lactate (at up to 1.5 μmol/min/gDW when the shuttle was inhibited completely). This suggests variable lactic acidosis seen in patients could depend on the capacity of a mechanism (such as the glycerol phosphate shuttle) to compensate for the extent of the deficiency. In the simulations, β-oxidation of fatty acids increased, which produced mitochondrial NADH and reduced the quinone pool via the electron transfer flavoprotein (ETF) and also compensated for the loss of acetyl-CoA needed for the TCA cycle (otherwise derived from the pyruvate created by glycolysis and lactate degradation).

#### 3.2.3 Complex I deficiency: compensation by cycling β-oxidation of fatty acids

Next flux through the putative proline cycle was limited (to 7.0 μmol/min/gDW), in addition to inhibiting the glycerol phosphate shuttle (Table S4). This predicted a further compensatory mechanism in the model with metabolite cycling by enzymes from the β-oxidation of fatty acids pathway (Fig. 2). In this hypothetical cycle, trans-2-enoyl-CoA reductase and acyl-CoA dehydrogenase were used together to oxidise NADPH and reduce the quinone pool. The NAPDH required came from three sources: nicotinamide nucleotide transhydrogenase (NNT) that converted NADH to NADPH with the translocation of a proton between compartments; glutamate dehydrogenase (GLDH) that degraded glutamate to oxoglutarate; and isocitrate dehydrogenase 2 (IDH2), that converted isocitrate to oxoglutarate. As the cycling of metabolites by fatty acid β-oxidation enzymes has not been experimentally characterised, this represented another model-generated hypothesis.

#### 3.2.4 Complex I deficiency: compensation by a folate shuttle

To uncover any remaining compensatory mechanisms in the model, the glycerol phosphate shuttle, NNT, and the proline cycle were all disabled (Table S5). This caused a major decrease of flux through the respiratory chain and consequent decrease of ATP production (30 μmol/min/gDW), suggesting only minor mechanisms remained. As complex I flux was reduced, pyruvate carboxylase converted pyruvate into oxaloacetate and then malate by reversing these steps of the TCA cycle. This removed mitochondrial NADH that otherwise could not be oxidised. Further pyruvate was converted into malate (via citrate and oxoglutarate) by using steps in the forward direction of the TCA cycle. Malate from both sources was then exported into the cytosol, which is consistent with organic acids accumulating in some complex I deficient patients (Dionisi-Vici et al., 1997, Esteitie et al., 2005). This bifurcation of the TCA cycle reduced its capacity to metabolise acetyl-CoA produced from β-oxidation of fatty acids. However, the cycling between metabolites catalysed by trans-2-enoyl-CoA reductase and acyl-CoA dehydrogenase continued. But as the supply of NADPH required for this was limited from the TCA cycle and amino acid degradation (since the produced oxoglutarate could not be metabolised), a new “folate shuttle” mechanism occurred whereby NADPH was supplied by coupling formate metabolism with tetrahydrofolate, the pentose phosphate pathway and the conversion of cytosolic malate into pyruvate (Fig. 2 and Supplementary Fig. 1). In this mechanism, NADPH was transported across the mitochondrial inner membrane by using tetrahydrofolate (THF) conversion reactions in conjunction with serine and glycine transport. This compensatory pathway predicted in the model overlaps with a characterised folate-dependant NADPH production pathway in which formate was speculated to be involved (Fan et al., 2014), although this shuttle has not been reported in complex I deficiency and the reversibility of the reactions needs to be experimentally verified under these conditions. The use of formate is unusual, but formate is produced in mitochondria and readily transported across membranes, albeit by an unknown transporter, as part of folate metabolism (Tibbetts and Appling, 2010). However, the model showed that the overall favourable gains in ATP production are limited by the ATP required by the reaction step that combines formate and THF to create 10-formyl-THF. Alternative entry points into this folate shuttle, replacing formate, may be possible from including folate-related reactions absent from the model.

#### 3.2.5 Complex I deficiency: metabolite compensation

Supply of certain metabolites may help compensate complex I deficiency. But, the cellular availability of metabolites is unknown in complex I deficiencies, and may differ to the model parameters that were taken from experimental measurements of healthy resting hearts. Therefore, we performed a series of simulations where the availability of particular metabolites was increased greatly, to explore their predicted affect on metabolism in complex I deficiency. We found maximum ATP production was increased in the model by supplying several amino acids—glutamate, arginine, proline, valine, aspartate, lysine and glutamine—due to their degradation and this occurred for all levels of complex I activity (Fig. 2 and Table S6). This increase was partially a consequence of increasing the supply of NADPH that could be used in the metabolite cycling reactions of fatty acid β-oxidation (Fig. 2). The degradation of these metabolites led to increased flux through the glutamate dehydrogenase reaction. Amino acid degradation also resulted in by-product excretion, including ammonia, alanine, lactate and oxaloacetate. In addition ATP production was increased in the model by supplying TCA cycle intermediates oxaloacetate, citrate and malate. Most effective was citrate, which increased ATP production by 16.5% with fully inhibited complex I, whereas oxaloacetate was least effective with maximum 1.7% increase at 50% of normal complex I flux, and was exported when the complex I flux dropped below 25% of normal (Table S7). Thus the model predicts dietary supply of several metabolites may increase cardiac ATP production in complex I deficiency.

A variety of different metabolic phenotypes occur in patients with complex I deficiency. The simulation results indicate this may arise from the different tissue-specific availabilities of amino acids and other metabolites that can boost ATP production. Cardiac catabolism of branched chain amino acids is crucial in ATP production and normal cardiac physiology (Huang et al., 2011), hence increased supply of these metabolites may improve the energetic state in both ischaemic heart and complex I deficiency (Drake et al., 2012). In addition, reservoirs of amino acid can temporally buffer hearts in adverse conditions such as ischemia (Drake et al., 2012). But although various metabolites improve cellular ATP production, there are problems associated with their supplementation. For instance, the degradation of many amino acids produces ammonium that is toxic, especially to the developing central nervous system (Braissant et al., 2013) where it can adversely alter several metabolic and signalling pathways, and cause depletion of mitochondrial ketoglutarate during its removal and thus impacting the TCA cycle. Further, complex I deficiency frequently has a neurological presentation and excess amino acids could cause signalling abnormalities and neurological damage, e.g. glutamate excitotoxicity (Lau and Tymianski, 2010). However, amino acids do not diffuse freely across the blood brain barrier (Smith, 2000). Complex I deficient patients have been placed on ketogenic diets with varying success (Roestenberg et al., 2012, Kanabus et al., 2016, Rahman, 2012). But although the ketone body acetoacetate was imported in simulations when complex I flux fell below 25% of its normal level, its contribution to ATP production was minimal (data not shown). Thus the model results suggest the improvement of some patients on ketogenic diets may not be due to increased ATP generation from ketone body degradation, but perhaps directly from increased dietary supply of fatty acids for β-oxidation or other mechanisms (Paoli et al., 2014).

### 3.3 Simulations of complex II deficiency

Complex II (succinate dehydrogenase) deficiencies are rare and account for about 2% of respiratory chain disorders (Loeffen et al., 2000, Rustin et al., 1993). Complex II oxidises succinate to fumarate whilst reducing ubiquinone to ubiquinol. This complex has a unique place in central metabolism as it connects the TCA cycle with the respiratory chain, but does not pump protons. Deficiencies are commonly associated with mutations in the catalytic *SDHA* subunit and *SDHAF1* assembly factor (Jain-Ghai et al., 2013), but also with mutations in catalytic *SDHB* and membrane-anchoring *SDHD* subunits (Alston et al., 2012, Jackson et al., 2014). Phenotypes of complex II deficiency largely overlap with those of complex I (Alston et al., 2012), and complex II inhibition commonly causes build-up of TCA cycle intermediates (Ohlenbusch et al., 2012, Jackson et al., 2014, Brockmann et al., 2002, Burgeois et al., 1992), but this is not a distinguishing diagnosis (Jackson et al., 2014, Sugimoto et al., 2000).

In our simulations of complex II deficiency, the model showed less ability to compensate compared to complex I deficiency, and ATP production fell from 138.1 to 66 μmol/min/gDW when complex II activity was inhibited completely (Table S8). This was due to the deficiency affecting both the respiratory chain and TCA cycle; fundamentally disrupting central metabolism into two ways. When we performed multiple simulations exploring different compensatory pathways it became clear that maximising ATP production in complex II deficiency required maximising NADH production while minimising the production of acetyl-CoA that fed into the TCA cycle.

#### 3.3.1 Complex II deficiency: compensation by the pentose phosphate pathway and a folate shuttle

**Fig. 3:**
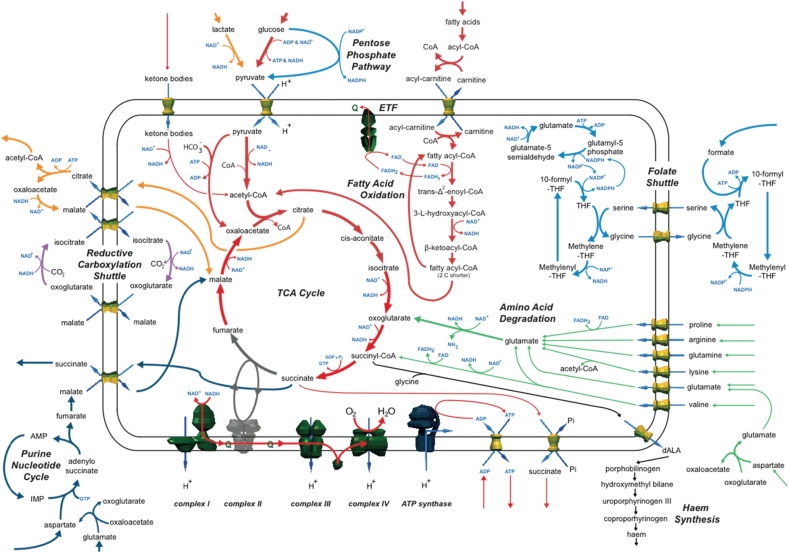
Summary of simulations on the effect of complex II deficiency on metabolism. As flux through complex II was inhibited in the simulations (grey), this affected both the TCA cycle and the respiratory chain and succinate efflux occurred when complex II flux was reduced by more than 10%. To compensate for complex II deficiency, the model predicted various pathways to produce cytosolic NADPH that was imported to the mitochondrion via either THF metabolism and a folate shuttle (blue) or a reductive carboxylation shuttle (purple). The mitochondrial NADPH was converted to NADH and used by complex I. The purine nucleotide cycle also contributed to a mechanism providing malate to bridge the broken TCA cycle (grey).

Initial simulations of complex II deficiency showed intermediates of glycolysis were diverted through the pentose phosphate pathway (Fig. 3 and Table S8). In addition, a large cytosolic flux converted citrate transported from the TCA cycle to oxoglutarate. Both of these pathways produced considerable quantities of cytosolic NADPH that entered a predicted folate shuttle (also seen in complex I deficiency and with the same caveats). However, in simulations of complex II deficiency the NADPH was converted to mitochondrial NADH and oxidised by complex I. To achieve this, the folate shuttle was coupled to an additional cycle that involved glutamate, glutamyl-5-phosphate and glutamate-5-semialdehyde (Supplementary Fig. 2). By using the pentose phosphate pathway in this way, NADH production was maximised while pyruvate production was minimised. This was important as the capacity of the TCA cycle to metabolise acetyl-CoA was restricted with complex II inhibited, and so this mechanism allowed the metabolism to continue of ketone bodies, leucine, isoleucine, cysteine, and β-oxidation of fatty acids. For complex II fluxes less than 90% of the normal value, the simulations predicted a large efflux of succinate (up to 1.7 μmol/min/gDW) – a common patient symptom. Further, the simulation predicted a shuttle allowing cytosolic NADH from lactate oxidation to cross the mitochondrial inner membrane while simultaneously producing malate to complete the TCA cycle. Thus lactate was reduced to pyruvate and the cytosolic NADH produced used to convert oxaloacetate to malate (Fig. 3). This was coupled to the production of oxaloacetate from citrate by cytosolic ATP citrate lyase (ACL), and required excess acetyl-CoA to be effluxed to a metabolite sink in the model (perhaps in vivo this would accumulate trapping CoA, or be metabolised by reactions not included in the model). The shuttle was completed in the model by counter exchange of cytosolic malate for mitochondrial citrate. A subsequent simulation showed that the ACL reaction was inactive when it was decoupled from oxaloacetate (Table S9), suggesting the disposal of acetyl-CoA from the TCA cycle at the cost of ATP production was unfavourable. In addition, to increase capacity of the TCA cycle to metabolise acetyl-CoA in the model, oxaloacetate was produced by pyruvate carboxylase and from malate generated from aspartate via the purine nucleotide cycle, and then imported into the mitochondrial matrix via counter-exchange with succinate (Fig. 3). This contribution of pyruvate carboxylase to maintain the TCA cycle in the simulations parallels its essentiality in supporting anabolism in paragangliomas that have inactive succinate dehydrogenase (Lussey-Lepoutre et al., 2015).

#### 3.3.2 Complex II deficiency: reductive carboxylation shuttle

To identify additional compensatory mechanisms in the model, the folate shuttle was disabled by preventing the uptake of formate (Table S10). This increased flux through the TCA cycle, but ATP production still fell (from 66.0 to 55.1 μmol/min/gDW at 0% complex II activity). The major change was the activity of another NADPH shuttle that used cytosolic reductive carboxylation (Fig. 3 and Supplementary Fig. 3) – whereby isocitrate and oxoglutarate were cycled on either side of the mitochondrial inner membrane. This was again dependent on the activity of the pentose phosphate pathway to deliver cytosolic NADPH. However, this process required the fixation of carbon dioxide, which will likely prevent or limit the flux that can be supported in vivo.

#### 3.3.3 Complex II deficiency: restricting succinate efflux

A key symptom of complex II deficiencies is organic acids accumulate in the blood and urine. In many patients succinate also accumulates as complex II is the major enzyme to metabolise it and cellular concentrations cannot be buffered (Briere et al., 2005). In some patients, oxoglutarate accumulates (Burgeois et al., 1992). Although succinate efflux was a persistent feature of our simulations, an efflux of oxoglutarate was not seen. To determine if this was a consequence of unrestricted succinate export in the simulations, we restricted succinate efflux to model its insufficient removal (Table S11). This resulted in oxoglutarate export from the model, matching the physiological phenotype, and also a very small flux through haem production (Fig. 3). This predicted haem flux is intriguing as mitochondrial iron-rich inclusion bodies have been reported in some complex II-deficient patients, but have been attributed to underlying iron metabolism disorders, such as impaired FeS cluster biosynthesis, causing abnormalities in the respiratory chain (Haller et al., 1991).

#### 3.3.4 Complex II deficiency: metabolite compensation

To predict the effect of increased metabolite availability on complex II deficiency, we individually simulated increased availability of different metabolites (Fig. 3 and Table S12). Glutamate, arginine, proline, valine, aspartate, lysine and glutamine provided notable compensation in simulations when complex II was completely inhibited, with maximum ATP production decreasing by between 2.5% to 21% depending on metabolite, compared to a 52% decrease in the simulations with default metabolite availabilities. This compensation was due to increased amino acid degradation producing mitochondrial NADH for complex I, and proline and valine degradation directly adding electrons to the quinone pool. The oxoglutarate produced via mitochondrial glutamate dehydrogenase entered the TCA cycle generating further NADH and GTP before being effluxed as succinate. These simulations suggest that maintaining ATP levels is partially determined by a tissue's ability to metabolise amino acids as the major source of energy and cope with metabolic by-products. This observation may explain the high degree of phenotype variability between different mitochondrial disease patients and variable tissue involvement.

### 3.4 Simulations of complex III and IV deficiencies

Complex III (ubiquinol:cytochrome *c* reductase) deficiencies are considered rare (Mourmans et al., 1997, Keightley et al., 2000), though may account for up to 15% of respiratory chain disorders (Loeffen et al., 2000, von Kleist-Retzow et al., 1998, Rustin et al., 1993). Complex III couples the redox reaction between ubiquinol and cytochrome *c* to proton pumping. In most deficiencies, mutations occur in either mitochondrial *MT-CYB* subunit or the nuclear *BCS1L* or *TTC19* assembly genes. For *BCS1L* mutations, common presentations include: muscle hypotonia, hepatopathy, renal tubulopathy, and organic acid accumulation (De Lonlay et al., 2001, Ramos-Arroyo et al., 2009). Mutations in *TTC19* have been noted to cause cerebral and cerebellar atrophy (Ghezzi et al., 2011, Morino et al., 2014, Nogueira et al., 2013). Whereas for *MT-CYB* mutations, presentations include: severe exercise intolerance, myoglobinuria and encephalopathies (Keightley et al., 2000). In some cases, alanine accumulation has been noted (Haut et al., 2003, Miyake et al., 2013).

Complex IV (cytochrome *c* oxidase) deficiencies account for about 28% of respiratory chain disorders (von Kleist-Retzow et al., 1998, Rustin et al., 1993). Complex IV is the final complex of the respiratory chain, and couples oxidation of cytochrome *c* to proton pumping, using oxygen as a terminal electron acceptor to produce water. Deficiencies associate with mutations in structural and assembly factors encoded on both nuclear and mitochondrial genomes (Shoubridge, 2001). Isolated complex IV deficiency is linked to mutations in nuclear-encoded assembly factors including *SURF1*, *SCO1*, *SCO2*, *COX10* and *COX15*. Clinical presentation is highly heterogeneous, however mutations in *SURF1* associate with Leigh syndrome; *COX10* with tubulopathy and leukodystrophy; and *SCO2* with encephalocardiomyopathy (Bohm et al., 2006, Valnot et al., 2000). Other common presentations of complex IV deficiencies include: aminoacidurias, lactic acidosis, hypotonia, and lipid accumulation in muscle fibres and liver, sometimes leading to myopathy and liver failure (von Kleist-Retzow et al., 1998, van Biervliet et al., 1977, Muller-Hocker et al., 1983, Lim et al., 2014).

For simulations of complex III and IV deficiencies, the predicted changes in fluxes of the metabolic pathways were the same (Table S13). This is because the two complexes function as a pair as they are neighbouring in the metabolic network with a single metabolite connecting them. As a result, inhibiting the flux of one complex in simulations consequently constrained the other. As the flux through these complexes was reduced in simulations, this reduced the flux through all the pathways contributing to the quinone pool, including complexes I and II, fatty acid β-oxidation, valine and proline degradation, and the glycerol phosphate shuttle. As a consequence, the model's ability to regenerate NAD^+^ was impaired restricting all other catabolic pathways, such as amino acid degradation and ketone body oxidation. As expected the only exception was glycolysis, which continued at all levels of inhibition, with any pyruvate unable to be metabolised by the TCA cycle effluxed as lactate. When the flux through either complex III or IV was completely abolished in the model, ATP production fell by 98% and almost all pathways were inactive. One of the few reactions still operating was mitochondrial malate dehydrogenase, which functioned in reverse to convert malate into fumarate. This fumarate was used as a terminal electron acceptor by complex II running in reverse to regenerate quinone from quinol and produce succinate, which was effluxed. This bypass of complex III and IV allowed a small flux through complex I to be maintained by a mechanism known as the NADH-fumarate reductase system (NFRS). Although this system is commonly seen in anaerobic bacteria and some intracellular parasites (Kita et al., 2002, Matsumoto et al., 2008, Kroger et al., 1992) it has been demonstrated in ischaemic human cardiomyocytes and requires a supply of fumarate (Chouchani et al., 2014). However, our simulations predicted its contribution to ATP production was minimal under default metabolite availability constraints.

As complex IV was the major consumer of oxygen in our model, simulating deficiencies in this part of the respiratory chain produced results similar to modelling ischemia (Chouchani et al., 2014). In this context, studies of fuel metabolism in ischaemic cardiac cells are especially relevant (Drake et al., 2012). Therefore, we investigated whether increasing the availability of amino acids boosted ATP production in the model (Table S14). But unlike with complex I and II, metabolite compensation had a minor effect on ATP production because the deficiencies ultimately blocked the whole oxidative metabolism of mitochondria. For example, when supplementing individual amino acids at arbitrarily high levels with 10% of normal complex III or IV flux, glutamine caused the greatest increase in ATP production, boosting it by 32%. But despite this increase, overall ATP production in the model was still only 16% of normal levels.

**Fig. 4:**
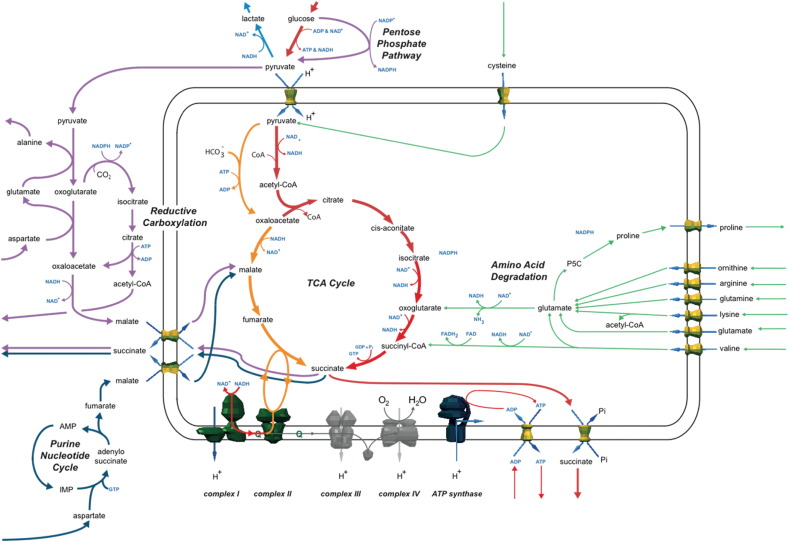
Summary of simulations on the effect of complex III and IV deficiency on metabolism. As maximum flux through either complex III or IV was reduced in the simulations (grey), there was a dramatic decrease in ATP production. Inhibiting complexes III and IV effectively blocks the respiratory chain. However, simulations predicted complex I continued pumping protons (and so supported the F-ATPase in producing ATP) if complex II ran in reverse and reduced fumarate to succinate—the NADH-fumarate reductase system (NFRS). Three pathways, which used a partial reversal of the TCA cycle, supplied the required fumarate for the NFRS: the purine nucleotide cycle, aspartate transaminase, and reductive carboxylation.

When the results of these simulations were combined into an overview (Fig. 4), it became clear the main end point of metabolism was succinate and this was the major metabolite effluxed. Many of the active pathways were the same as seen in simulations of complex I and II deficiencies, with additional ATP/GTP and NADH produced by the degradation of glutamate, glutamine, arginine, ornithine, and valine. However, as in the model the ability to use NADH and quinone declined as the inhibition of complex III and IV increased, the contribution from these pathways was steadily restricted. Unique to these simulations was the NFRS, which was enhanced by the availability of additional metabolites. Three pathways, which used a partial reversal of the TCA cycle, supplied the required fumarate for the NFRS: the purine nucleotide cycle, aspartate transaminase, and reductive carboxylation (Supplementary Fig. 4). These pathways had the added benefit of regenerating NAD^+^ or avoiding production of NADH. In simulations with glutamine and 5% or lower flux activity of complex III and IV, proline biosynthesis was predicted to become active and used as an additional pathway to remove NADH with the produced proline effluxed from the model.

Although these simulations predict the NFRS is not useful for increasing ATP production directly in complex III or IV deficiency, there may be indirect benefits, such as supporting the proton motive force across the mitochondrial inner membrane to maintain mitochondrial viability. One key difference between these simulations and those of ischemia is the in vivo role of succinate accumulation. In ischemia, succinate is responsible for ischaemic reperfusion injury due to its rapid metabolism by complex II when oxygen is reintroduced (Chouchani et al., 2014). This causes reverse electron transport of electrons from succinate to complex I resulting in a burst of damaging reactive oxygen species. This is not an issue with complex III and IV deficiency, but it is unknown whether the accumulation of succinate has other effects.

## 4 Conclusions

We have shown computer models of mitochondrial metabolism can simulate known features of respiratory chain disorders, supporting their biological applicability. The model's comprehensive coverage enabled prediction of multiple pathways that have the potential to improve the energetic state of diseased mitochondria and cells. Thus we show computer modelling can generate hypotheses about compensatory pathways and by their testing provide new opportunities for future research on mitochondrial disease. Further, modelling and simulation can also provide a framework to interpret the data from large-scale ‘omics experiments on mitochondrial dysfunction.

Simulations of complex I deficiency showed the smallest reduction in ATP production, and predicted three metabolic mechanisms that compensate for the deficiency. A proline cycle and cycling of fatty acid β-oxidation reactions allowed additional electrons to enter the respiratory chain via the quinone pool, in addition to the well-characterised glycerol phosphate shuttle. A folate cycle provided additional NADPH to the mitochondrial matrix to drive the fatty acid cycling. This predicted metabolic flexibility may explain the high proportion of isolated complex I disorders among respiratory chain diseases, because patients have the highest chance of survival via alternate compensating metabolic pathways.

Simulations of complex II deficiency showed a much larger drop in ATP production than complex I deficiencies because of the dual impact on the TCA cycle and respiratory chain. In the model this impaired the use of the main cellular fuel sources (glucose, fatty acids and ketone bodies), as the acetyl-CoA produced could not be fully metabolised. This made the model more reliant on alternative mechanisms to maintain complex I activity, such as a predicted folate and reductive carboxylation shuttles to take cytosolic NADPH produced from the pentose phosphate pathway and convert it to NADH in the mitochondrial matrix. The lack of pathways found in the model to compensate for complex II deficiency may explain the rarity of patients with complex II deficiency, perhaps due to embryo lethality. It may also imply that compensatory shuttles identified in the simulations cannot support the necessary fluxes in vivo, as also suggested by their predicted inefficiency in producing ATP.

Simulations of deficiencies of complex III and IV both had the same effect on metabolism and lacked alternative compensatory pathways and metabolites. The changes were strikingly similar to experimentally validated simulations of ischemia (Chouchani et al., 2014). Although the model predicted activity of the NADH–fumarate reductase system—an unusual mechanism that uses fumarate as an alternative terminal electron acceptor to oxygen—its predicted impact on ATP production was minimal, even with a notable increase in the availability of amino acid substrates. But, residual activity of complex III and IV is high in most patients, and so decreases in ATP production will perhaps not be as dramatic as predicted by the simulations of low enzyme flux, perhaps explaining the large number of patients documented. However, embryos with more severe complex III and IV deficiencies are likely unviable, dying in utero, and thus are not observed.

Certain amino acids commonly were predicted to increase ATP production in simulations of all respiratory chain disorders. It is possible that the plethora of symptoms in mitochondrial diseases arise from differences in the substrates that are available to different tissues and in different patients. The model we used was based on a cardiomyocyte mitochondrion with default parameters to reflect the wide range of substrates cardiomyocytes can access. The reactions included in this model are likely shared with many other tissue types. But the substrate availabilities are likely different among tissues, thus limiting the compensatory options available to some tissues and placing them at greater risk of energy deficiency.

In the future, the quality of simulation results and compensation strategies could be improved and personalised by measuring and incorporating data from patients about metabolite changes, expression of metabolic enzymes, metabolic fluxes, and regulatory interactions. We anticipate our work can guide future experimental studies that can confirm or reject the hypothetical mechanisms revealed by computer modelling and their importance in mitochondrial respiratory chain disorders. We hope that our results will promote the use of computational modelling by the mitochondrial diseases community and will spur investigation into new research directions and therapies for mitochondrial respiratory diseases, such as changes in diet or the provision of adjuvants to boost anaplerotic pathways.

The following are the supplementary data related to this article.

Supplementary File 1 SBML-formatted file of the flux balance analysis model used for the simulations. Supplementary File 1

Supplementary tables. Calculated fluxes of reactions during simulations of the models under control conditions, mitochondrial dysfunction, and with the addition of selected metabolites. Image 1

Supplementary Fig. 1 The contribution of a folate shuttle to ATP production in simulations of complex I deficiency. During simulations of complex I deficiency, a folate shuttle emerged (blue) to support ATP production when the compensatory mechanisms of the glycerol phosphate shuttle, NNT and proline cycle were disabled. This folate shuttle used enzymes of tetrahydofolate metabolism to shuttle NADPH produced from the pentose phosphate pathway into the mitochondrial matrix. This NADPH was used to drive the cycling of metabolites between trans-2-enoyl reductase (purple) and acyl-CoA dehydrogenase (red), reducing the quinone pool and contributing to flux through the respiratory chain to produce ATP. Supplementary Fig. 1

Supplementary Fig. 2 The contribution of a folate shuttle to ATP production in simulations of complex II deficiency. Initial simulations of complex II deficiency showed a folate shuttle that used enzymes from tetrahydofolate metabolism to shuttle NADPH produced from the pentose phosphate pathway into the mitochondrial matrix (blue), as seen previously in simulations of complex I deficiency. However, in complex II deficiency this was coupled to an additional cycle involving glutamate to convert NADPH into NADH that was then oxidised by complex I. This reduction of the quinone pool contributed to flux through the respiratory chain to produce ATP. Supplementary Fig. 2

Supplementary Fig. 3 Other shuttles contributing to ATP production in simulations of complex II deficiency. A. Initial simulations of complex II deficiency showed the citrate malate antiporter used to shuttle cytosolic NADH into the mitochondrion, which was oxidised by complex I. B. A reductive carboxylation shuttle emerged (purple) during simulations of complex II deficiency when the folate shuttle (Supplementary Fig. 2) was disabled. This shuttled NADPH produced by the pentose phosphate pathway into the mitochondrial matrix as NADH, which was oxidised by complex I. Supplementary Fig. 3

Supplementary Fig. 4 Sources of fumarate for the NADH-fumarate reductase system in simulations of complex III & IV deficiency. During simulations of deficiencies of complex III and complex IV, the NADH-fumarate reductase system (NFRS) (orange) emerged as a mechanism to support ATP production. The NFRS uses complex II running in reverse with fumarate as the terminal electron acceptor. For the necessary fumarate, the model used a series of reactions to convert pyruvate into malate and then fumarate (purple). Supplementary Fig. 4

## Author contributions

AJR conceived the study. AJR and LZ designed the simulation procedures. LZ and AGS conducted the simulations. ACS developed and provided the model. LZ and ACS analysed the results and drafted the manuscript. All authors reviewed, edited and approved the final manuscript.

## Conflict of interests

The authors declare that they have no conflict of interest.
