# Supplementary figures and images for: Metabolic flexibility of mitochondrial respiratory chain disorders predicted by computer modelling
Source: Mitochondrion. 2016 Nov;31:45–55. doi: 10.1016/j.mito.2016.09.003 (PMC5115619; doi:10.1016/j.mito.2016.09.003)

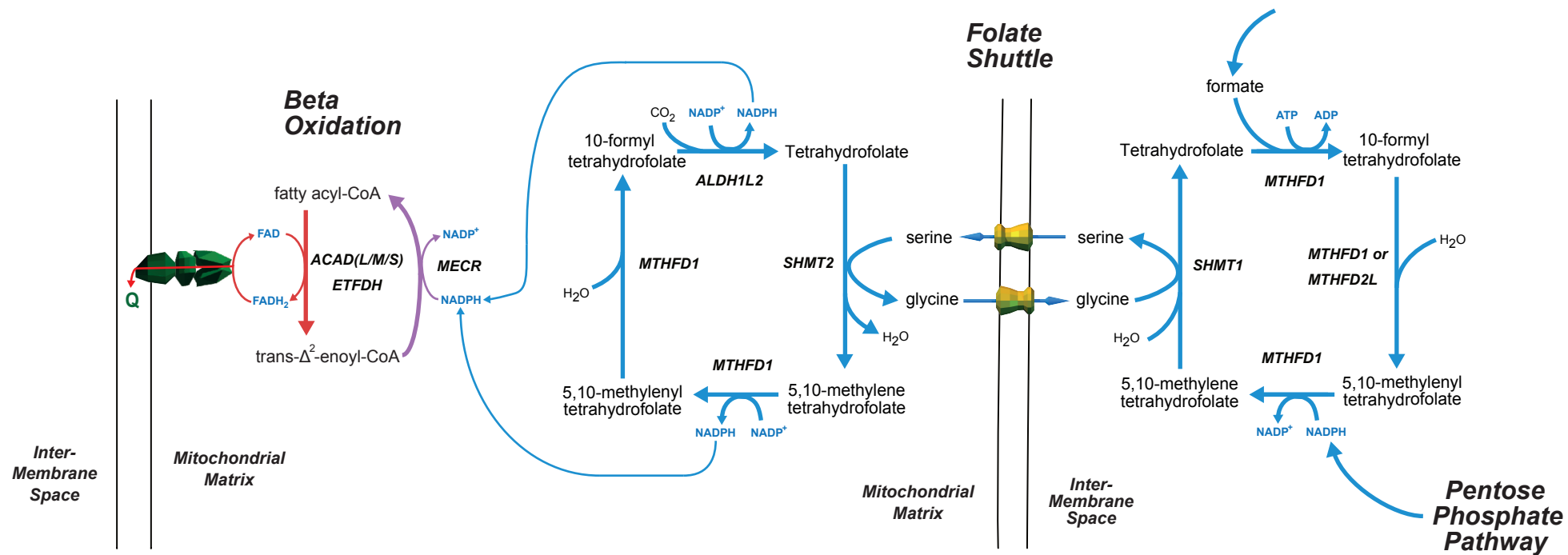

Supplement: Supplementary Fig. 1 — The contribution of a folate shuttle to ATP production in simulations of complex I deficiency. During simulations of complex I deficiency, a folate shuttle emerged (blue) to support ATP production when the compensatory mechanisms of the glycerol phosphate shuttle, NNT and proline cycle were disabled. This folate shuttle used enzymes of tetrahydofolate metabolism to shuttle NADPH produced from the pentose phosphate pathway into the mitochondrial matrix. This NADPH was used to drive the cycling of metabolites between trans-2-enoyl reductase (purple) and acyl-CoA dehydrogenase (red), reducing the quinone pool and contributing to flux through the respiratory chain to produce ATP. [file mmc3.pdf]

# Folate Shuttle

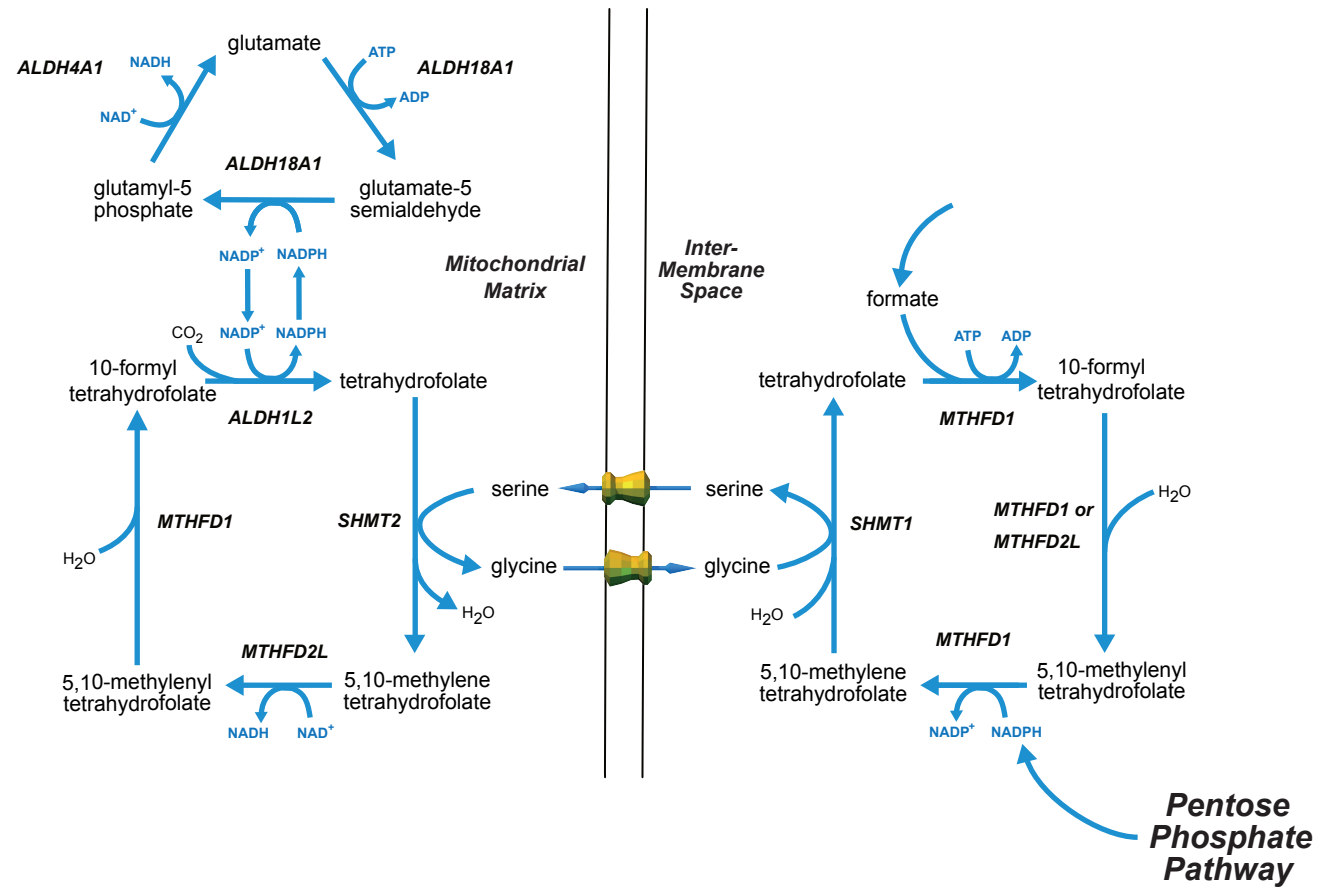

Supplement: Supplementary Fig. 2 — The contribution of a folate shuttle to ATP production in simulations of complex II deficiency. Initial simulations of complex II deficiency showed a folate shuttle that used enzymes from tetrahydofolate metabolism to shuttle NADPH produced from the pentose phosphate pathway into the mitochondrial matrix (blue), as seen previously in simulations of complex I deficiency. However, in complex II deficiency this was coupled to an additional cycle involving glutamate to convert NADPH into NADH that was then oxidised by complex I. This reduction of the quinone pool contributed to flux through the respiratory chain to produce ATP. [file mmc4.pdf]

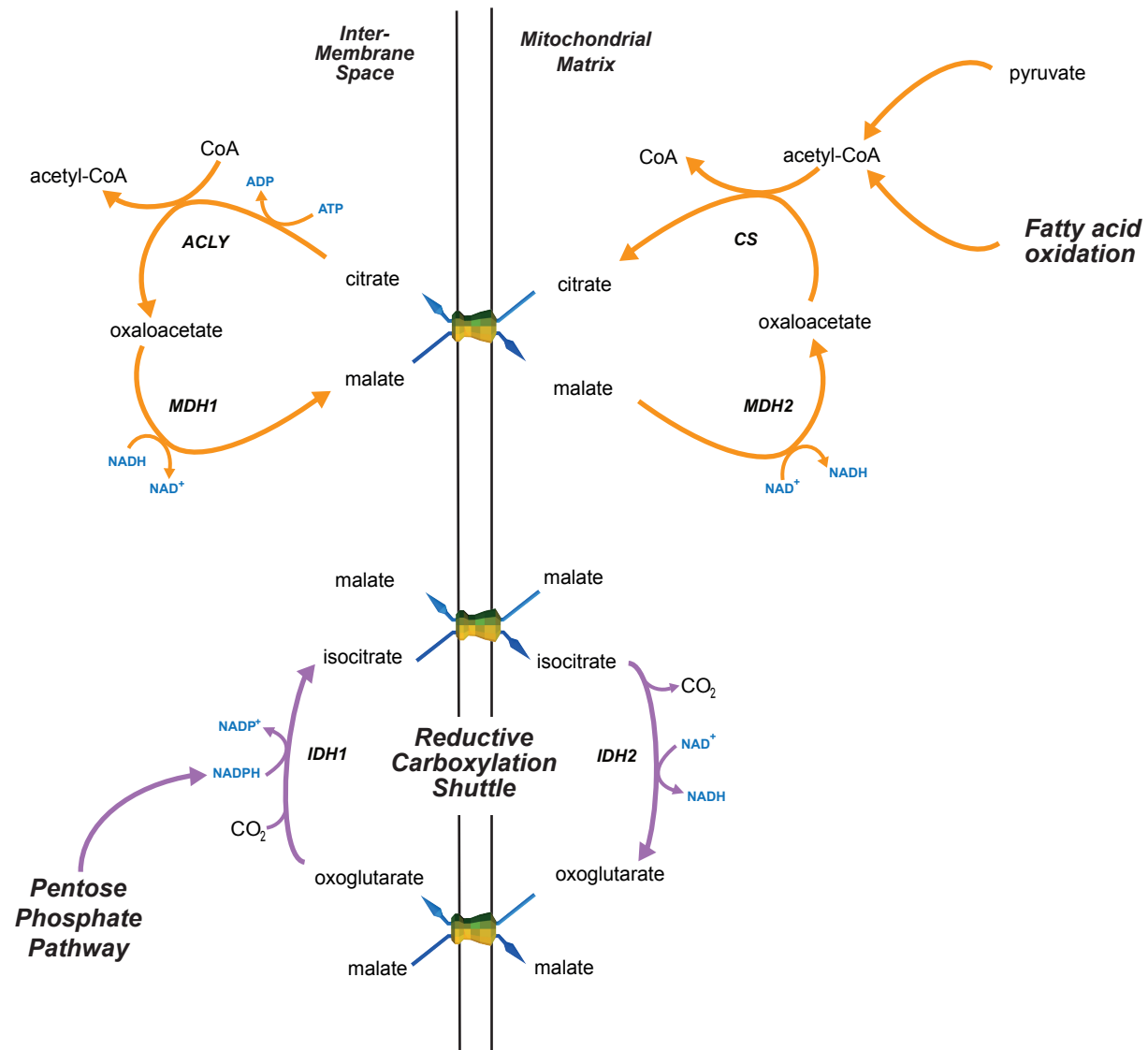

Supplement: Supplementary Fig. 3 — Other shuttles contributing to ATP production in simulations of complex II deficiency. A. Initial simulations of complex II deficiency showed the citrate malate antiporter used to shuttle cytosolic NADH into the mitochondrion, which was oxidised by complex I. B. A reductive carboxylation shuttle emerged (purple) during simulations of complex II deficiency when the folate shuttle (Supplementary Fig. 2) was disabled. This shuttled NADPH produced by the pentose phosphate pathway into the mitochondrial matrix as NADH, which was oxidised by complex I. [file mmc5.pdf]

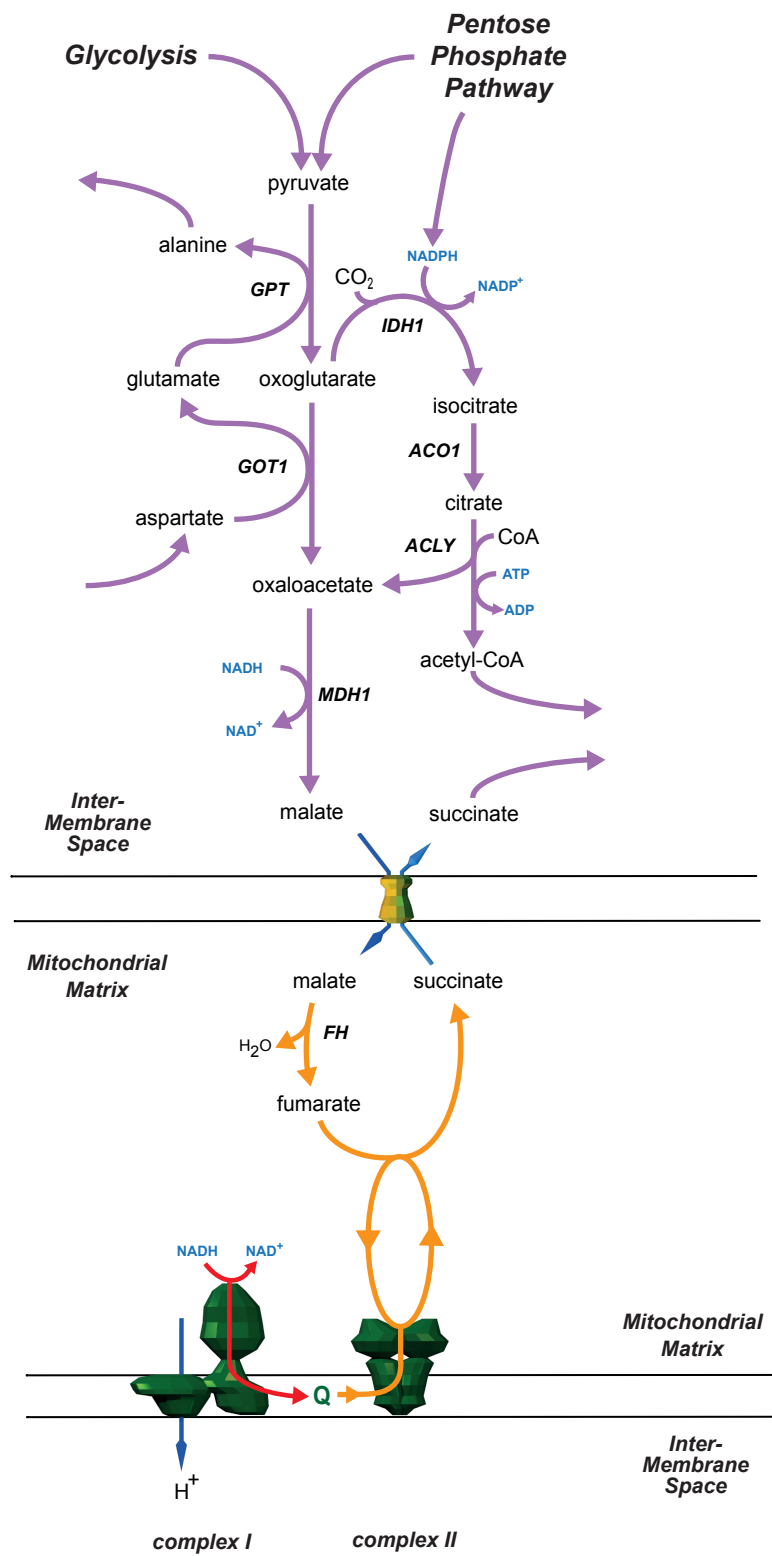

Supplement: Supplementary Fig. 4 — Sources of fumarate for the NADH-fumarate reductase system in simulations of complex III & IV deficiency. During simulations of deficiencies of complex III and complex IV, the NADH-fumarate reductase system (NFRS) (orange) emerged as a mechanism to support ATP production. The NFRS uses complex II running in reverse with fumarate as the terminal electron acceptor. For the necessary fumarate, the model used a series of reactions to convert pyruvate into malate and then fumarate (purple). [file mmc6.pdf]
